# Comparison of outcomes from sepsis between patients with and without pre-existing left ventricular dysfunction: a case-control analysis

**DOI:** 10.1186/cc13840

**Published:** 2014-04-23

**Authors:** Daniel R Ouellette, Sadia Z Shah

**Affiliations:** 1 Pulmonary and Critical Care Medicine, Henry Ford Hospital, K-17, 2799 West Grand Blvd, Detroit, MI 48202, USA

## Abstract

**Introduction:**

The aim of this study was to determine if there are differences between patients with pre-existing left ventricular dysfunction and those with normal antecedent left ventricular function during a sepsis episode in terms of in-hospital mortality and mortality risk factors when treated in accordance with a sepsis treatment algorithm.

**Methods:**

We performed a retrospective case-control analysis of patients selected from a quality improvement database of 1,717 patients hospitalized with sepsis between 1 January 2005 and 30 June 2010. In this study, 197 patients with pre-existing left ventricular systolic dysfunction and sepsis were compared to 197 case-matched patients with normal prior cardiac function and sepsis.

**Results:**

In-hospital mortality rates (*P* = 0.117) and intubation rates at 24 hours (*P* = 0.687) were not significantly different between cases and controls. There was no correlation between the amount of intravenous fluid administered over the first 24 hours and the PaO_2_/FiO_2_ ratio at 24 hours in either cases or controls (r^2^ = 0.019 and r^2^ = 0.001, respectively). Mortality risk factors for cases included intubation status (*P* = 0.016, OR = 0.356 for no intubation), compliance with a sepsis bundle (*P* = 0.008, OR = 3.516 for failed compliance), a source of infection other than the lung (*P* = 0.019, OR = 2.782), and the initial mixed venous oxygen saturation (*P* = 0.004, OR = 0.997). Risk factors for controls were the initial platelet count (*P* = 0.028, OR = 0.997) and the serum lactate level (*P* = 0.048, OR = 1.104). Patients with pre-existing left ventricular dysfunction who died had a lower initial mean mixed venous oxygen saturation than those who survived (61 ± 18% versus 70 ± 16%, *P* = 0.002).

**Conclusions:**

Clinical outcomes were not different between septic patients with pre-existing left ventricular dysfunction and those with no cardiac disease. There was no correlation between fluid administration and oxygenation at 24 hours in either cohort. The mortality risk factor profile of patients with pre-existing left ventricular dysfunction was different when compared with control patients, and may be related to oxygen delivery determinants.

## Introduction

Severe sepsis and septic shock represent serious medical conditions with high morbidity and mortality rates. Sepsis is a leading cause of death [1,2], and frequently causes end-organ damage [3], as well as cognitive and physical disability in survivors [4]. In the United States, over $14 billion were spent on hospitalizations for sepsis in 2008 alone [5]. The hospitalization rate for sepsis has more than doubled between 2000 and 2008, with a rate of 24 per 10,000 persons in 2008 [2]. Patients hospitalized with sepsis have a length of stay nearly twice that of other patients and are eight times more likely to die than other patients [2,6]. Early aggressive treatment for sepsis increases the chances for survival [7]. Tenets of aggressive treatment include early antibiotic therapy [8] and aggressive resuscitative strategies to achieve monitored clinical goals [9].

Patients with sepsis are a heterogeneous group with various predisposing features and sources of infection. Patients who are critically ill from all causes have a higher mortality when they have cardiac dysfunction [10]. Sepsis may be associated with concurrent reversible left ventricular dysfunction (LVD) [11] that some authors have attributed to circulating factors [2,12]. Patients with LVD that occurs during sepsis may have a worse prognosis than patients who do not have this condition [12,13]. Cardiac disease has a high prevalence in hospitalized patients [14], which may complicate the management of sepsis. However, the outcomes for septic patients who have pre-existing LVD have not been carefully studied.

Early sepsis management requires aggressive fluid resuscitation to defined clinical endpoints, whereas it may be necessary to be more circumspect in the administration of fluids in later phases of sepsis [15]. The presence of LVD in critically ill septic patients complicates this strategy and leads to concerns about the development of pulmonary edema and respiratory failure from excessive fluid resuscitation. Alterations in the cardiac compliance in patients with LVD may diminish the utility of the central venous pressure as a preload assessment tool [16]. Both the mixed venous oxygen saturation (ScVO_2_) and the serum lactate level may be affected by the presence of LVD as well as sepsis, potentially confounding their utility in patients with both disorders [17-19]. More data are needed to guide clinicians in the management of these difficult patients.

Our institution has maintained an extensive database since 2005 for all hospitalized patients treated for sepsis. We selected a population of patients with both acute sepsis and prior evidence of LVD for this study. In order to determine if differences exist between septic patients with pre-existing LVD and those without, we selected case-matched control patients with normal pre-sepsis LV function for comparison purposes. We chose to analyze clinical parameters of both case and control patients in order to identify risk factors for in-hospital mortality. The identification of differences between septic patients with and without pre-existing LVD, and the ascertainment of sepsis mortality risk factors in each group, may lead to the refinement of management algorithms in the subpopulation of patients with pre-existing LVD.

## Materials and methods

### Study design and enrollment

All patients at our institution who developed sepsis after January 2005 had medical data entered into an institutional sepsis quality improvement database. Patients admitted to the Emergency Department, hospital wards or intensive care units with a diagnosis of sepsis, as well as those who developed sepsis during the course of their hospital stay for another diagnosis, were included in the database. We retrospectively reviewed data collected between 1 January 2005 and 30 June 2010. We additionally examined data from the institutional electronic medical record (EMR) of all patients enrolled in the sepsis database. The study was performed at a quaternary health care system located in the midwestern United States. The study protocol was ethically reviewed and approved by the Henry Ford Health Systems Institutional Review Board (IRB project number 6237; Henry Ford Health Systems Institutional Review Board). The need for informed consent was waived by the Henry Ford Health Systems Review Board because the study was an observational non-interventional retrospective study using a database where patient identification information had been removed.

Case subjects were identified as those patients entered into the sepsis database that had an echocardiogram performed and entered into the EMR prior to (but not more than two years before) the incidence of sepsis with a reported left ventricular ejection fraction (LVEF) of 50% or less. When patients had multiple echocardiograms reported in the EMR prior to the sepsis event, we used the results from the echocardiogram performed most proximate to the event. Each case subject had the diagnosis of sepsis confirmed by retrospective review of the EMR. If a potential case subject had multiple admissions entered into the database, only the first admission was considered for study purposes. Potential case subjects were excluded if they were receiving renal replacement therapy prior to the sepsis event. These patients were excluded because the physiologic factors associated with end-stage renal disease and its management could confound the analysis of the effects of left ventricular dysfunction in patients with sepsis. Patients with any degree of renal insufficiency not requiring renal replacement therapy (including patients who had received a kidney transplant), or who developed a need for renal replacement therapy while being treated for sepsis, were included in the study if they met enrollment criteria.

Control subjects were chosen from the database for each case subject in a 1:1 ratio by selecting from age- and gender-matched patients with an LVEF greater than 50% by echocardiogram (not concurrent but within two years of the sepsis event). The potential control subject with a date of birth closest to the case subject was selected. Potential control subjects were excluded if they had cardiac wall motion abnormalities, valvular cardiac abnormalities, significant evidence of diastolic dysfunction on echocardiogram, or had any evidence of an acute cardiac ischemic syndrome during their hospitalization for sepsis. Control subjects were otherwise required to meet the same inclusion criteria as case subjects. The information for each study subject was entered into a research database without patient identifiers for analysis.

### Definitions

“Sepsis” was defined as being present if a patient manifested at least two of four systemic inflammatory response syndrome criteria and had documented evidence of infection. “LVEF” was defined as the estimated fractional diminution in size of the left ventricular cavity during systole as reported in standard echocardiographic interpretations. When a range was provided in a report for LVEF, the midpoint of that range was chosen as the LVEF for study purposes. “Sepsis bundle” was defined as the algorithm used for sepsis management described by Rivers and co-workers [9]. “Compliance with the sepsis bundle” was defined as being present when the patient was treated completely in accordance with the sepsis bundle. The patient was not compliant with the sepsis bundle if any parameter of the sepsis bundle was not met. “Time 0” was defined as the point in time when the sepsis bundle was initiated for each study subject. “Intubation” was defined as endotracheal intubation and mechanical ventilation within 24 hours of Time 0.

### Statistical analysis

We performed all statistical analysis using SPSS software version 18 with a logistical regression add-on package version 20 (International Business Machines, Armonk, New York, United States of America) and considered *P* <0.05 to be statistically significant unless otherwise specified. We used chi-squared tests, two-sample *t* tests, or Mann-Whitney U tests as appropriate for univariable analysis. Adjustments were not made for multiple comparisons.

For both case and control subjects, we analyzed the relationship between the dependent variable PaO_2_/FiO_2_ at 24 hours and the total intravenous fluid volume administered over 24 hours using linear regression analysis. Results are displayed as scatter plots and the goodness of fit is indicated by r^2^.

Cases and controls were matched for age and gender. We used multivariable logistic regression analysis with mortality as the dependent variable to additionally control for the effects of ScVO_2_, hematocrit, lactate levels and intubation status on the association of mortality between cases and controls.

We developed a model to identify risk factors for mortality by multivariable logistic regression analysis in both the case and control subject populations. Potential risk factors were identified from a univariable analysis of each of the available variables using mortality as the dependent variable. Variables were selected for analysis if they were significantly associated with mortality (*P* <0.1) and if at least 80% of the case and control subjects had data available for the variable. A correlation matrix was constructed for the continuous independent variables in the case and control subject populations and the variables were examined for collinearity by Pearson’s correlation.

## Results

Clinical data were collected in a quality improvement database at our institution for 1,717 hospitalized patient episodes with a diagnosis of sepsis between 1 January 2005 and 30 June 2010. Of these patients, 510 died during their hospital stay, while 1,207 survived until discharge, for an in-hospital all-cause mortality rate of 29.7%. We identified 197 study subjects who met inclusion criteria and had both sepsis and pre-existing LVD. We chose 197 control subjects with normal left ventricular function from the quality improvement database matched to cases according to age and gender. Comparisons between the mean values for various clinical variables for cases and controls are presented in Table 1. Comparisons of clinical outcomes for cases and controls are presented in Table 2. When controlled for ScVO_2_, hematocrit, lactate levels and intubation status by multivariable logistic regression analysis using mortality as the dependent variable, the mortality risk between cases and controls was not different (*P* = 0.183, OR = 1.41). The other risk factors studied, ScVO_2_ (*P* = 0.005, OR = 0.998), intubation (*P* = 0.000, OR = 2.56), hematocrit (*P* = 0.012, OR = 0.996) and initial lactate level (*P* = 0.037, OR = 1.078) were all significantly associated with mortality.

**Table 1:** Comparisons of clinical variables between cases and controls

**Variable**	**Cases (n)**	**Controls (n)**	***P***
Age (years)	68 +/- 16 (197)	67 +/- 16 (197)	0.935^b^
Male gender	129 (197)	129 (197)	1.000^a^
LVEF (%)	35 +/- 12 (197)	60 +/- 5 (197)	**<0.001**^**b**^
APACHE II	20 +/- 7 (194)	19 +/- 8 (191)	0.603^b^
Initial CVP (cm H_2_O)	9 +/- 8 (128)	8 +/- 7 (120)	0.334^b^
ScVO_2_ (%)	67 +/- 17 (166)	71 +/- 16 (166)	**0.009**^**b**^
Lung Infection	64 (197)	54 (197)	0.322^a^
Sepsis bundle compliant	61 (197)	69 (197)	0.453^a^
MAP <65 mm Hg	116 (197)	114 (197)	0.919^a^
Initial temperature (°C)	36.9 +/- 1.6 (196)	37.0 +/- 1.5 (195)	0.714^b^
Temperature at 24 hours (°C)	37.2 +/- 2.0 (197)	37.3 +/- 2.0 (197)	0.471^b^
Initial heart rate (min^−1^)	104 +/- 27 (197)	108 +/- 24 (197)	0.074^b^
Heart rate at 24 hours (min^−1^)	115 +/- 32 (197)	117 +/- 29 (197)	0.360^b^
Initial respiratory rate (min^−1^)	24 +/- 9 (197)	24 +/- 9 (197)	0.558^b^
Respiratory rate at 24 hours (min^−1^)	29 +/- 9 (197)	30 +/- 9 (197)	0.435^b^
Initial MAP (mm Hg)	74 +/- 22 (197)	76 +/- 23 (197)	0.502^b^
MAP at 24 hours (mm Hg)	58 +/- 19 (197)	60 +/- 21 (197)	0.226^b^
Initial pH	7.35 +/- 0.12 (176)	7.35 +/- 0.13 (175)	0.994^b^
pH at 24 hours	7.31 +/- 0.13 (112)	7.29 +/- 0.15 (107)	0.302^b^
Initial PO_2_/FiO_2_	259 +/- 130 (161)	266 +/- 139 (166)	0.630^b^
PO_2_/FiO_2_ at 24 hours	265 +/- 113 (108)	268 +/- 126 (115)	0.846^b^
Initial sodium (mg/dL)	139 +/- 7 (196)	139 +/- 12 (197)	0.730^b^
Initial creatinine (mg/dL)	3.04 +/- 2.72 (195)	2.73 +/- 3.28 (197)	0.115^c^
Initial hematocrit (%)	31.6 +/- 6.6 (197)	33.1 +/- 8.1 (197)	**0.046**^**b**^
Initial WBC (x10^3^ ul)	15.1 +/- 9.7 (196)	16.4 +/- 15.6 (197)	0.294^b^
Initial platelet count (x10^3^ ul)	234 +/- 138 (195)	231 +/- 153 (196)	0.844^b^
Initial lactate (mmol/L)	3.4 +/- 3.0 (196)	4.1 +/- 3.5 (195)	**0.019**^**c**^
Initial glucose (mg/dL)	136 +/- 98 (197)	136 +/- 52 (197)	0.960^b^
Initial 24-hour fluid volume (ml)	4,005 +/- 2,763 (190)	4,378 +/- 3,040 (192)	0.321^c^
Receipt of vasoactive agents	88 (197)	79 (197)	0.415^a^

Significance, P <0.05; ^a^Chi-square test; ^b^Student’s *t*-test; ^c^Mann-Whitney U test. APACHE II, Acute Physiology and Chronic Health Evaluation II; CVP, central venous pressure; LVEF, left ventricular ejection fraction; MAP, mean arterial pressure; PO_2_/FiO_2_, partial pressure of oxygen to the fraction of inspired oxygen; ScVO_2_, mixed venous oxygen saturation; WBC, white blood count.

**Table 2:** Comparison of clinical outcomes between cases and controls

**Variable**	**Cases (n)**	**Controls (n)**	***P***
MAP >65 mm Hg following fluid resuscitation	101 (197)	114 (197)	0.225
MAP >65 mm Hg following receipt of vasoactive agents for hypotension	74 (85)	64 (75)	0.532
Initiation of dialysis	2 (197)	1 (197)	1.000
Intubated	93 (197)	98 (197)	0.687
Mortality	63 (197)	48 (197)	0.117

Chi-square test used for dichotomous variables. MAP, mean arterial pressure.

**Figure 1:**
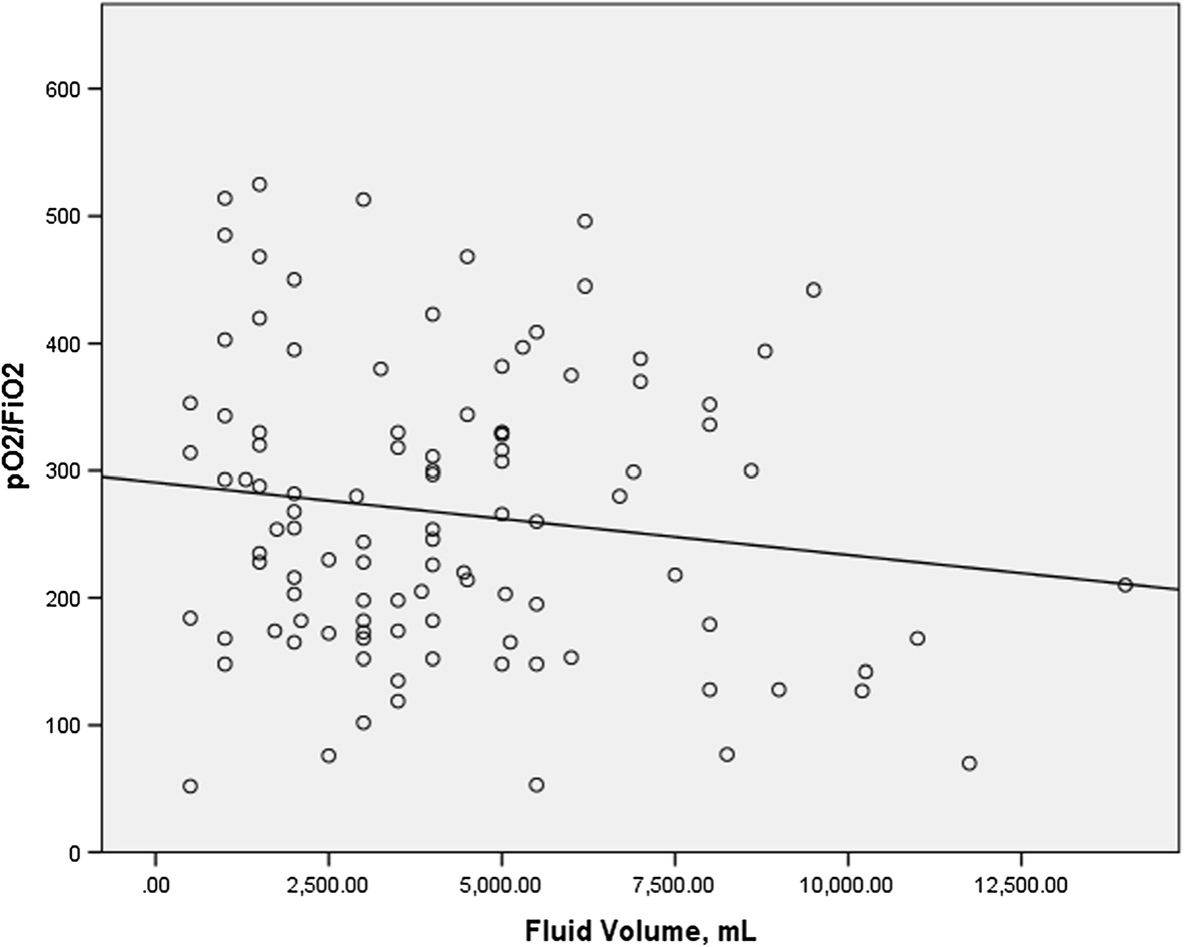
**Intravenous fluid volume over the initial 24 hours versus PO2/FiO2 at 24 hours for cases.** r^2^ = 0.019.

**Figure 2:**
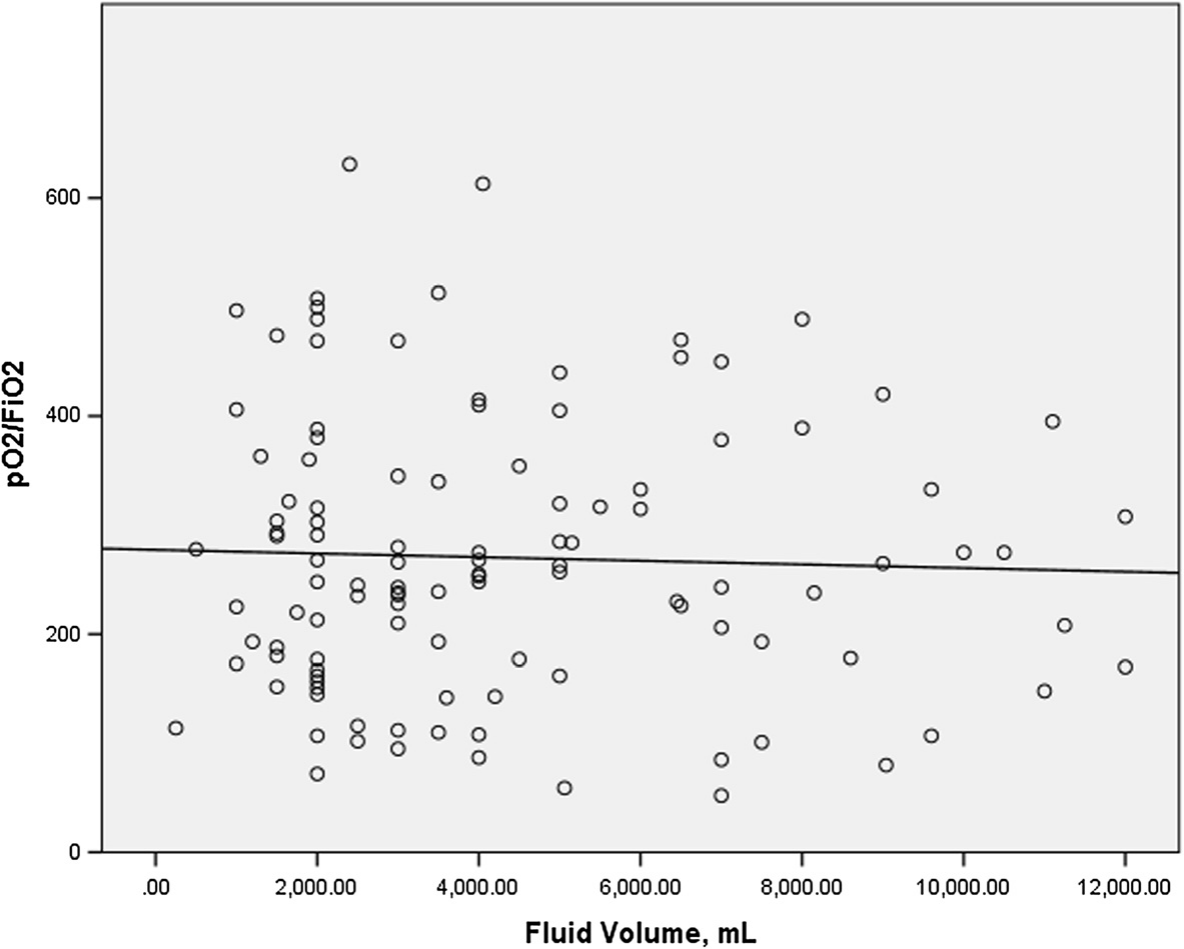
**Intravenous fluid volume over the initial 24 hours versus PO**_**2**_**/FiO**_**2 **_**at 24 hours for controls.** r^2^ = 0.001.

In order to study the effect of the administration of intravenous fluid volume on respiratory gas exchange, we examined the relationship between the total intravenous fluid volume administered during the first 24 hours of patient admission with the ratio of the partial pressure of oxygen to the fraction of inspired oxygen (PO_2_/FiO_2_) obtained at 24 hours by linear regression analysis in both cases and controls. Data for the volume of fluid administered were available for most patients (n = 190 for cases; n = 192 for controls), but the data availability for the PO_2_/FiO_2_ at 24 hours was more limited (n = 108 for cases; n = 115 for controls). We found no relationship between intravenous fluid volume and the PO_2_/FiO_2_ at 24 hours in either cases or controls (r^2^ = 0.019 for cases, r^2^ = 0.001 for controls; Figures 1 and 2).

We examined the population of patients with pre-existing LVD to determine if there were differences between those subjects with more severe LVD compared to those subjects with less severe dysfunction other than the reduction in LVEF. There were 81 patients with an LVEF of less than 35%, compared to 116 patients with an LVEF greater than or equal to 35% but less than 50%. Patients with an LVEF less than 35% received less intravenous fluid during their resuscitation than did patients with a higher LVEF (3.40+/-2.07 L compared with 4.42+/-3.09 L, *P* = 0.012). However, mortality rate (27% versus 35%, *P* >0.05), intubation rate (47% versus 43%, *P* >0.05), use of vasopressors (46% versus 44%, *P* >0.05), Acute Physiology and Chronic Health Evaluation II (APACHE II) score (20+/-7 versus 19+/-6, *P* = 0.285), ScVO2 (67.9+/-16.0% versus 66.0+/-17.1%, *P* = 0.472), and initial lactate levels (3.8+/-3.7 mmol/L versus 3.1+/-2.5 mmol/L, *P* = 0.121) were not statistically different between patients with an LVEF of less than 35% and those with an LVEF between 35% and 50%.

We studied both the case and the control populations to determine the risk factors for mortality in each group. We applied univariable analysis of 25 potential risk factors with mortality as the dependent variable to both the cases and control groups. Results are presented in Table 3. Significant correlations by Pearson’s correlation were demonstrated in both populations between the APACHE II score and the lactate level (*P* = 0.001). We chose to exclude APACHE II from the multivariable model. We also found a significant correlation between the hematocrit and respiratory rate in the case but not the control subjects (*P* = 0.003 for the cases, *P* = 0.210 for the controls). We chose to exclude respiratory rate from the model as well. The model used for the multivariable logistic regression analysis, with mortality as the dependent variable, and which was applied to the case and control population in turn, included the following covariates: intubation status, ScVO_2_, initial hematocrit, initial platelet count, compliance with a sepsis bundle, lung infection, serum lactate level and the use of vasoactive agents to augment blood pressure. The results of this analysis are presented in Table 4. Risk factors for mortality were different between cases and controls. Significant risk factors for mortality among patients with pre-existing left ventricular dysfunction included the ScVO_2_, compliance with a sepsis algorithm, intubation status and a source of infection other than the lung. Significant risk factors for mortality among patients with pre-existing normal left ventricular function included the initial platelet count and the initial lactate level. While the ScVO_2_ was similar between survivors and non-survivors among control patients, the ScVO_2_ was significantly lower among non-surviving patients with pre-existing LVD compared with surviving patients (Table 5).

**Table 3:** Risk factors for mortality among cases and controls by univariable analysis

**Risk factors**	***P*, cases (n)**	***P*, controls (n)**
Age	0.122 (197)	0.199 (197)
Initial CVP	0.232 (128)	0.731 (120)
Initial temperature	0.210 (196)	0.243 (195)
Initial heart rate	0.792 (197)	0.885 (197)
Initial respiratory rate	0.626 (197)	**0.01** (197)^*^
Initial MAP	0.792 (197)	0.636 (197)
Initial PO_2_/FiO_2_	0.254 (161)	0.267 (166)
PO_2_/FiO_2_ at 24 hours	0.155 (108)	**0.013** (115)^*^
Initial pH	0.374 (176)	0.432 (175)
pH at 24 hours	**0.001** (112)^*^	**0.028** (107)^*^
Initial creatinine	0.166 (195)	0.466 (197)
Initial hematocrit	**0.074** (197)^*^	**0.071** (197)^*^
Initial WBC	0.619 (196)	0.672 (197)
Initial platelet	0.974 (195)	**0.055** (196)^*^
APACHE II	**0.028** (194)^*^	**0.007** (191)^*^
Initial lactate	**0.008** (196)^*^	**0.019** (195)^*^
24-hour fluid volume	0.576 190)	0.747 (192)
ScVO_2_	**0.002** (166)^*^	0.882 (168)
Glucose level	0.717 (197)	0.136 (197)
No vasoactive drug use	**0.016** (197)^#^	**0.009** (197)^#^
No Intubation	**0.001**(197)^#^	**0.007** (197)^#^
LVEF	0.236 (197)	0.319 (197)
Fail bundle compliance	**0.002** (197)^#^	0.328 (197)
Gender	0.811 (197)	0.880 (197)
Source other than lung	**0.006** (197)^#^	0.754 (197)

Student’s *t*-test^*^ and Chi Square^#^; *P* <0.1 significant. APACHE II, Acute Physiology and Chronic Health Evaluation II; CVP, central venous pressure; LVEF, left ventricular ejection fraction; MAP, mean arterial pressure; PO_2_/FiO_2_, partial pressure of oxygen to the fraction of inspired oxygen; ScVO_2_, mixed venous oxygen saturation; WBC, white blood count.

**Table 4:** Risk factors for mortality among cases and controls by multivariable logistic regression analysis

**Risk factors**	***P*, cases**	**Odds ratio, cases**	***P*, controls**	**Odds ratio, controls**
No intubation	0.016*	0.356	0.081	0.474
Initial hematocrit	0.148	0.996	0.091	0.996
Initial platelet	0.700	0.999	0.028*	0.997
Fail bundle compliance	0.008*	3.516	0.760	0.872
Source other than lung	0.019*	2.782	0.539	0.773
Initial lactate	0.490	1.040	0.048*	1.104
No vasopressors	0.641	1.228	0.071	0.463
ScVO_2_	0.004*	0.997	0.239	0.999

*P* <0.05 is significant*. ScVO_2_, mixed venous oxygen saturation.

**Table 5:** **Comparison of initial mean ScVO**_**2 **_**between survivors and non-survivors, cases and controls**

**Group**	**n**	**ScVO**_**2 **_**(%)**	***P***
Non-survivors, cases	57	61+/-17	
Survivors, cases	109	70+/-16	0.002^a^
Non-survivors, controls	44	71+/-15	
Survivors, controls	124	72+/-16	0.882^b^

^a^Comparison of means by Student’s *t*-test, non-survivors and survivors, cases.

^b^Comparison of means by Student’s *t*-test, non-survivors and survivors, controls. ScVO_2_, mixed venous oxygen saturation.

## Discussion

To our knowledge, this is the first study to compare patients with sepsis and pre-existing LVD to patients with sepsis with no significant pre-existing cardiac abnormalities. We found that septic patients with pre-existing LVD differed only modestly from patients with preserved left ventricular function at our institution. Both populations had similar degrees of severity of illness, as assessed by the APACHE II score. Although patients with pre-existing LVD had significantly lower ScVO_2_ values, hematocrit levels and initial lactate levels than did patients with normal pre-sepsis ventricular function, other demographic and laboratory determinants were similar. Important clinical outcomes, such as in-hospital mortality and intubation rates, were not significantly different between the two groups of patients, though there was a trend towards increased mortality in the patients with pre-existing LVD.

Fluid management in critically ill patients with sepsis is complex, in part because the clinical endpoints for fluid administration change during the course of the septic episode. Initially, there is a general consensus that fluid administration should be liberal and aggressive [5,9]. Clinical management during the initial 24 hours of therapy emphasize the use of resuscitation goals that implicate sufficient tissue delivery of oxygen and include not only traditional hemodynamic measurements and urine output but also variables related to oxygen delivery and consumption, such as hemoglobin and mixed venous oxygen saturation. Subsequently, however, patient outcomes are improved in critically ill septic patients by observing conservative fluid management strategies [20]. The supporting rationale for the application of conservative fluid management strategies during the late stages of sepsis stem from observations of improved patient outcomes from the use of such strategies for the treatment of acute lung injury [21,22]. Complex patients with both pre-existing LVD and sepsis might be perceived as being more susceptible than other septic patients to developing respiratory complications from aggressive initial fluid resuscitation because pulmonary edema is a component of the congestive heart failure syndrome. Although patients with an LVEF below 35% did not have a different severity of illness nor different outcomes than patients with LVD but with higher LVEF, they did receive statistically less intravenous fluid during this resuscitation. We speculate that this may reflect practice patterns directed by the assumption just noted. We observed that patients with both sepsis and pre-existing LVD were no more likely to be intubated and require mechanical ventilation than case-matched controls, although the need for intubation was a mortality risk factor in these patients. Additionally, we found no correlation between the amount of resuscitative fluid administered over the first 24 hours and oxygenation in that group of cases and controls for whom data were available. We suggest that patients with sepsis and pre-existing LVD may be no more likely than other patients to develop respiratory compromise from goal-directed volume resuscitation in early sepsis.

Studies involving animal models over 40 years ago demonstrated that serum lactate levels were elevated when tissue oxygenation was decreased by experimental manipulation of the determinants of oxygen delivery [23,24]. Increased lactate levels have been demonstrated to be associated with poor outcomes in critically ill patients [25], patients with circulatory shock [26], and patients with severe sepsis [27]. Production of lactate appears to be increased both in patients with circulatory shock and sepsis, though the putative mechanisms may be different: in circulatory shock mechanisms implicating oxygen delivery play an important role, whereas in sepsis constitutive cellular mechanisms of lactate production are prominent [28]. While there are some data that suggest that lactate clearance may be preserved in circulatory shock [29], lactate clearance seems to be diminished in some patients with sepsis [30]. We observed that the mean initial lactate level for patients who had sepsis and pre-existing LVD was less than that of the control group in our study, though the mean lactate levels for both groups were elevated compared to normal values. Initial lactate levels were closely associated with mortality in our septic control patients, but not in our septic patients with pre-existing LVD. The reasons for these findings are not clear, but may be related to the complex interaction of multiple factors effecting lactate metabolism in patients with multi-system disease. We suggest that initial serum lactate levels may be less important a predictor of outcome in septic patients with pre-existing LVD than they are in other septic patients.

Kasnitz and co-workers studied the relationship between ScVO_2_ and lactate levels in patients with cardiopulmonary disease, noting that ScVO_2_ levels were decreased in such patients and associated with increased lactate levels and mortality [17]. The presumed mechanism for this phenomenon is that diminished cardiac output leads to decreased oxygen delivery to tissues, increased oxygen extraction and, therefore, diminished oxygen saturation in blood returning to the heart [31]. In a large cohort of patients undergoing coronary artery bypass graft surgery, a threshold value for ScVO_2_ less than 60.1% was identified below which mortality was increased [32]. In contrast to the findings in patients with cardiogenic shock, early work suggested that ScVO_2_ was normal or increased in patients with septic shock [33]. Mechanistically, it has been suggested that this phenomenon occurs because of decreased oxygen extraction and utilization. Krafft and co-workers provided evidence supporting this hypothesis in general, but suggested that short-term decrements in ScVO_2_ might be associated with adverse clinical outcomes [34]. More recently, Rivers and associates demonstrated that a strategy that, in part, targets augmenting ScVO_2_ in septic patients leads to improved clinical outcomes [9]. However, the control group in the latter study had a mean ScVO_2_ of 49%, which is different from that expected from prior work. Concerns have been raised about the ability to generalize the findings of Rivers *et al*. given that most septic populations have ScVO_2_ levels in the normal or supranormal range [35].

We found that patients with pre-existing LVD and sepsis had a lower mean initial ScVO_2_ than a control group of septic patients, a finding which is supported by previous work [36]. Patients with pre-existing LVD in our study who died had a significantly lower ScVO_2_ than survivors, and low ScVO_2_ was an independent risk factor for mortality. These findings were not observed in the control cohort. The additional observation that failure to comply with a protocol targeted to improve oxygen delivery variables was an independent risk factor for mortality in cases, but not controls, suggests to us that patients with pre-existing LVD may have a different physiological response profile during sepsis than other septic patients. We postulate that sepsis is a heterogeneous disorder, and management strategies that target oxygen delivery variables may be particularly appropriate for patients with pre-existing LVD.

Our work suffers from a number of important limitations. This study is from a single center, which may limit the applicability of the results. We retrospectively reviewed a quality improvement database; so that there is a risk that unappreciated confounding variables may be present. We did not assess left ventricular function systematically during the sepsis event in any patients, leading to the possibility that controls or cases may have had unrecognized synchronous LVD. The number of patients included in our study was relatively small, limiting the strength of our conclusions. Larger, prospective studies will need to be performed to confirm our findings.

## Conclusions

This single center retrospective study demonstrates that patients with sepsis and pre-existing LVD may have similar outcomes when compared to patients with sepsis without pre-existing LVD. No correlation was demonstrated between the amount of intravenous fluid administered over the first 24 hours and oxygenation at 24 hours in septic patients either with or without pre-existing LVD. Mortality risk factors for patients with pre-existing LVD and sepsis included a low ScVO_2_, failure to comply with a sepsis bundle of therapies, intubation status, and a source of infection other than the lung, whereas mortality risk factors for septic patients with no previous evidence of cardiac disease included increased lactate levels and a low platelet count. Further study will be needed to determine if improvement of oxygen delivery during sepsis is more important in ameliorating outcome in patients with pre-existing LVD compared to those without.

## Key messages

•Patients with sepsis and pre-existing left ventricular dysfunction have similar outcomes when compared to patients with sepsis with normal pre-existing left ventricular function.

•Patients with pre-existing left ventricular dysfunction who suffer from sepsis may be no more likely than patients with normal left ventricular function and sepsis to develop respiratory compromise when treated aggressively with intravenous fluids.

•Patients with pre-existing left ventricular dysfunction who develop sepsis have different mortality risk factors than other patients. Mortality risk factors are related to oxygen delivery variables in these patients.

## Abbreviations

APACHE II: Acute Physiology and Chronic Health Evaluation II; CVP: Central venous pressure; EMR: Electronic medical record; LVD: Left ventricular dysfunction; LVEF: Left ventricular ejection fraction; OR: Odds ratio; PO2/FiO2: Partial pressure of oxygen/Fraction of inspired oxygen; ScVO2: Mixed venous oxygen saturation; WBC: white blood count.

## Competing interests

Both authors are salaried employees of Henry Ford Health Systems in Detroit, MI, USA. Henry Ford Health System patients and infrastructure were utilized in this research, and the health care system may benefit from an enhanced reputation by the publication of this article. Henry Ford Health System may pay publishing costs of this work. The authors have no other competing interests to declare.

## Authors’ contributions

Both DRO and SS made substantial contributions to the study’s conception and design, were involved in acquiring data, and participated in analyzing and interpreting the data. Both DRO and SS were involved in writing and revising the manuscript in terms of important intellectual content. Both DRO and SS have given approval for manuscript publication. Both authors read and approved the final manuscript.

## Authors’ information

DRO is a Senior Staff Physician in the Pulmonary and Critical Care Medicine Division at Henry Ford Hospital and is an Associate Professor of Medicine at Wayne State University School of Medicine. SS is a Fellow in Pulmonary and Critical Care Medicine at Henry Ford Hospital.
